# Illness Attitudes Associated with Seasonal Depressive Symptoms: An Examination Using a Newly Developed Implicit Measure

**DOI:** 10.1155/2015/397076

**Published:** 2015-12-10

**Authors:** Katherine Meyers, Michael A. Young

**Affiliations:** Department of Psychology, Illinois Institute of Technology, Chicago, IL 60616, USA

## Abstract

The Dual Vulnerability Model of seasonal depression posits that seasonal vegetative symptoms are due to a physiological vulnerability, but cognitive and mood symptoms are the result of negative appraisal of vegetative changes. In addition, rumination may be associated with stronger negative attitudes toward vegetative symptoms. This is the first study to examine implicit attitudes toward vegetative symptoms. We hypothesized that illness attitudes about fatigue moderate the relationship between the severity of vegetative symptoms and the severity of cognitive symptoms and that the illness attitudes are associated with rumination. This study also developed an implicit method to assess the appraisal of fatigue as indicating illness. Results supported both hypotheses. Illness attitudes toward fatigue moderated the relationship between vegetative symptoms and cognitive symptoms. Ruminative response style was positively associated with implicit illness attitudes towards fatigue. The study provides support for the role of negative appraisals of vegetative symptoms in the development of cognitive and mood seasonal depressive symptoms.

## 1. Introduction

Seasonal affective disorder (SAD) consists of annually recurring depressive episodes that begin in the fall/winter and remit in the spring/summer. It is diagnosed in the 5th edition of the Diagnostic and Statistical Manual of Mental Disorders (DSM-5) [1] as recurrent Major Depressive Disorder with a seasonal specifier. The DSM definition allows seasonal patterns to occur at any time of the year and a relatively rare summer depression pattern has been observed [2]. In this paper we will use the terms SAD and seasonality to refer strictly to the winter pattern, a usage common in the field. Individuals with SAD usually experience marked vegetative symptoms (e.g., fatigue, appetite increase and weight gain, carbohydrate craving, and hypersomnia) in addition to cognitive and affective depressive symptoms such as sadness, difficulty in concentrating, and feelings of worthlessness or hopelessness [3, 4]. Seasonal vegetative symptoms alone have been shown to occur to varying degrees in the general population [3, 5, 6] and are not limited to diagnosable seasonal affective disorder.

Researchers have observed that the onset of seasonal vegetative symptoms precedes the onset of the cognitive and affective depressive symptoms [7–9]. The Dual Vulnerability Model of Seasonal Depression [9, 10] was proposed to explain this pattern of depressive symptomatology. The model proposes that SAD is the result of the combination of two separate vulnerabilities. The first is a physiological vulnerability for seasonal vegetative symptoms in the wintertime. The second is a psychological vulnerability for cognitive and mood symptoms in response to physiological changes. In this way, vegetative change serves as a stressor which triggers the latent diathesis of depressive vulnerability. Consequently, cognitive and affective symptoms develop after the onset of vegetative symptoms in those with the psychological vulnerability.

Support for the distinction between vegetative and cognitive/affective symptoms includes factor analyses of seasonal symptoms demonstrating that the clusters of symptoms emerge as two factors ([11–13]; see [14] for a different two-factor structure). Also, as would be predicted from the Dual Vulnerability Model, White and Terman [15] observed a subset of individuals who show vegetative changes but lack cognitive and mood symptoms, that is, exhibit a physiological vulnerability only. Finally, Young and colleagues (2008) found that vegetative symptoms interact with cognitive vulnerabilities to predict cognitive and mood SAD symptoms, as would be expected from a diathesis-stress model. Given that both winter vegetative symptoms and cognitive vulnerabilities to depression vary in severity across the general population, the model is applicable to a full range of seasonal symptomatology and is not limited to diagnosable seasonal affective disorder.

Research examining attitudes associated with vegetative seasonal symptoms can help elucidate the proposed cognitive processes linking vegetative symptoms to cognitive and mood symptoms. Previous studies examining the cognitions and attitudes associated with SAD have used self-report methods [16, 17]. However, self-report data are susceptible to biases due to limited insight, social desirability, fear of stigma, and other demand characteristics. Implicit tests are thought to be less influenced by such factors [18–21]. In contrast to self-report, implicit tests are based on a participant's performance on a discrimination task being influenced by the magnitude of his or her association between two concepts embedded in the task. This implicit association is thought to represent a memory or schema-based relationship between the concept of interest and a descriptive attribute (e.g., good or bad). Measuring attitudes implicitly is particularly useful because it can reduce biases associated with explicit measures and may give a more accurate representation of attitudes before a deliberative thought process takes place.

There are no existing implicit measures that assess implicit attitudes towards specific physiological or psychological symptoms. This study developed an implicit measure of attitudes toward fatigue, one of the most common seasonal vegetative symptoms and one for which we considered it possible that an individual's attitudes might increase the likelihood of developing psychological symptoms. Many implicit measures involve examining a construct that is bipolar (e.g., black-white, self-others). However, these measures are not useful when the category of interest, in this case the symptom of fatigue, is unipolar and does not have a natural counterpart. In this situation, the use of a single-category implicit measure is necessary. The Go/No-Go Association Task (GNAT) [22] was chosen for this study. Furthermore, implicit measures usually use a bipolar evaluative or attributional construct that is based on valence (e.g., good versus bad) or self-reference (e.g., me versus not me). However, this study examined a bipolar construct based on health (healthy versus ill), believed to represent a maladaptive cognition associated with vegetative symptoms in SAD (e.g., “my symptom of fatigue means I am ill”). Based on the Dual Vulnerability Model, we hypothesized that implicit illness attitudes towards fatigue moderate the relationship between the severity of winter vegetative symptoms and the severity of winter cognitive/mood symptoms. That is, we expected that although seasonal cognitive/affective symptoms may be associated with seasonal vegetative symptoms, the degree to which this is the case depends on the extent to which one associates his/her vegetative symptoms (i.e., feeling tired) with being ill (versus healthy). If this relationship holds true, attitudes or beliefs toward symptoms themselves could be a key factor in differentiating individuals who have clinically significant winter depressive symptoms among those with some degree of seasonality.

In addition, we hypothesized that illness attributions toward vegetative symptoms are associated with certain depressive cognitive styles, in particular, a ruminative response style. The Response Style Theory of depression [23], originally developed in reference to unipolar depression, also has been associated with seasonal symptomatology [10, 16, 24]. According to Response Style Theory, rumination is a response to distress characterized by thinking repetitively and passively about one's symptoms and their possible causes and consequences. Nolen-Hoeksema and Morrow [23] posited that one way in which the ruminative response style maintains depressed mood is by activating negative self-evaluations and attributions. That is, rumination makes negative attitudes and schemas more accessible, leading to more negative automatic thoughts and, in turn, to exacerbated and prolonged distress [25].

Ruminating on the experience, causes, and consequences of vegetative symptoms may exacerbate negative attitudes and lead to more severe cognitive symptoms and in turn depressed mood. In terms of the Dual Vulnerability Model, this cognitive style may act as a cognitive vulnerability that differentiates individuals with vegetative symptoms who develop seasonal affective disorder from those with vegetative symptoms who do not. Evidence supporting the relationship between rumination and seasonal depression includes findings that a ruminative response to depressed mood predicts the severity of depressive symptoms in the wintertime [24] and that rumination acts as a moderator of the relationship between daily vegetative and daily cognitive/mood symptoms in college students [10, study 1] and between weekly vegetative and weekly cognitive/mood symptoms in SAD sufferers [8]. In addition, in a study of SAD patients, Young et al. [10, study 2] found not only that daily fatigue predicted mood symptoms but also that rumination about fatigue moderated the relationship between the fatigue and low mood. Furthermore, Rohan and colleagues [16] found that individuals with a history of SAD had higher levels of trait rumination compared to age, gender, and education matched controls. Thus, the literature supports that rumination plays an important role in maintaining negative thoughts and attitudes and may be associated with more negative attitudes on a discrimination task like the GNAT.

In summary, research examining attitudes associated with vegetative symptoms of seasonal affective disorder may help elucidate the relationship between vegetative symptoms and cognitive/affective symptoms. Furthermore, rumination may be a cognitive style associated with relatively more negative attributions towards seasonal symptoms. Finally, assessing attitudes implicitly should help reduce potential bias associated with more traditional, direct ways of examining attitudes. Accordingly, we hypothesized that implicit illness attitudes toward seasonal vegetative symptom (represented by fatigue) moderate the relationship between seasonal vegetative symptoms and seasonal cognitive/mood symptoms. Additionally, we hypothesized that a more illness-related interpretation of symptoms is positively associated with a greater ruminative response style.

## 2. Methods

### 2.1. Participants and Procedures

Participants were 32 undergraduate students (21 female and 11 male) at a mid-size, Midwestern university. The study was described in psychology classes and students were offered extra credit for their participation. Potential participants were asked to participate only if they were fluent in English. The mean (SD) age of the participants was 21.3 (4.4) years (median = 20; range: 18–40). Participants were informed that the research was examining people's experiences with seasonal changes and that these exist on a continuum of severity. After giving informed consent, participants individually completed the Tired GNAT on a laptop computer. To prevent carryover effects, questionnaires, in a computerized format, were administered after the GNAT. Data were collected in April and May to avoid the effect of possible psychomotor retardation in the winter on response times. All procedures were approved by the Illinois Institute of Technology Institutional Review Board.

### 2.2. Measures

The Seasonality Assessment Form (SAF) [13] is a self-report questionnaire designed to assess a respondent's typical severity of seasonal depressive symptoms during the winter. Respondents rated the severity of each of 14 symptoms on a five-point Likert scale (0 indicating no change and 4 indicating severe change). Six items reflect vegetative symptoms (e.g., feel drowsy and crave sweets or carbohydrates) and six items reflect cognitive and affective symptoms (e.g., feeling sad or down and feeling inadequate or worthless). The advantages of this scale are that a full range of vegetative, cognitive, and affective symptoms is assessed and a Vegetative Seasonality Subscale (VSS) and a Psychological Seasonality Subscale (PSS), as well as a Total Seasonality Score (TSS), are generated; higher scores indicate more severe seasonal symptomatology. The VSS, PSS, and TSS have demonstrated good internal consistency (*α* ≥ .90) and convergent validity [13]. Coefficient alphas of the VSS and PSS in the current study were .79 and .89, respectively.

The five-item brooding subsection of the Ruminative Response Scale of the Response Style Questionnaire (RRS) [23, 26] was used to assess ruminative response style. The full scale consists of 22 questions that assess an individual's tendency to think about the causes and consequences of depressive symptoms. Participants indicate the degree to which they engage in a cognitive or behavioral response on a 4-point Likert scale, ranging from 0 (“almost never”) to 4 (“almost always”). The RRS has demonstrated good internal consistency (*α* = .90) and five-month test-retest reliability (*r* = .80) [27]. Cronbach's alpha for the brooding subscale in the current study was .71.

### 2.3. Implicit Attitudes toward Fatigue

The Tired Go/No-Go Association Task (GNAT) [21] was used to measure implicit illness attributions toward the vegetative symptom of fatigue. The GNAT was designed to determine the relative ease with which participants responded to fatigue-related stimuli when paired with illness-related stimuli compared to when paired with health-related stimuli. Although similar to the implicit associations test [19], the GNAT is useful when the target category (i.e., a depressive symptom) does not have a clear contrasting category (e.g., man-woman).

In this study, Tired GNAT word stimuli were presented rapidly in the center of a computer screen. Stimuli appeared on the screen until the participant responded, for a maximum of 1200 ms. There was a blank screen for 850 ms between stimuli. Participants were instructed to press the space bar (a “go” trial) when a stimulus word belonged to either of the two attribute categories in the upper corners of the screen: the target category “tired” and one of the two evaluative categories, “ill” or “healthy.” Participants were instructed not to respond if the stimulus did not belong to one of the two categories presented (a “no-go” trial). Stimuli consisted of words representing fatigue, health, or illness, neutral distractor words, and distractor terms consisting of other symptom words (hunger) to ensure that participants were focused on the symptom of fatigue (see Appendix). The mean (SD) number of letters per word was as follows: fatigue words, 7.25 (1.67), healthy words, 6.50 (2.77), and illness words, 7.25 (2.44). These word length differences were not statistically significant:* F*(3, 28) = 1.25 and *p* = .31. Data for analysis consisted of the response latency for each on each trial. The experiment was conducted using E-Prime 2.0 [28].

Participants were told that they would be categorizing words “as quickly as possible” and that they would first examine the words belonging to each category. Next, each of the primary lists of words and their category label (tired, healthy, and ill) were displayed for twenty seconds and participants were instructed to study the words as belonging to the category. This allowed participants to increase their familiarity with the word stimuli being used in the study and to decrease any uncertainty about which category words belonged to. This step was important because the point of the task is to assess the impact of category pairings on response time, not the participants' ability to discriminate the categories.

The Tired GNAT itself consisted of two practice blocks of trials and two experimental blocks of trials. The two practice blocks required discriminations unrelated to our hypotheses and were used to increase familiarity with the stimuli and the procedure in general. Each practice block consisted of 26 trials (16 fatigue, health, and illness terms and 10 distractor terms). The first practice block involved responding only to fatigue or hunger words and the second one involved responding only to healthy or ill words. For practice trials, feedback was given in the form of a red “X” presented on screen when a participant responded incorrectly.

For the experimental blocks (tired/healthy and tired/ill), the evaluative category term for the first block (healthy or ill) varied randomly across participants and then switched to the opposite term for the second block. Participants were instructed to respond as quickly as possible to stimuli that belong to “go” categories (tired and healthy/ill). In each experimental block, there were 32 go trials (8 tired words and 8 ill words, each presented twice) and 32 no-go trials (11 distractor words and 5 hungry terms, each presented twice) for a total of 64 trials per block. Order of word presentation was randomized.

Tired GNAT *D* scores were computed using response latency, as data suggest it is more reliable than response errors [22]. GNAT *D* scores are similar conceptually to Cohen's *d* [19] and compare response latencies of tired-ill trials and tired-healthy trials with shorter response latencies representing a greater association. *D* score for an individual is generated by computing the difference in mean response times between the tired-ill and tired-healthy blocks and dividing it by the standard deviation of response latencies for the participant across both blocks. (Following complete exclusion of the 3 cases with greater than 40% invalid trials, remaining invalid responses were imputed prior to computing *D* scores so that mean response times in each block were based on the same set of target words. This issue is relevant to other implicit tests but has not been recognized in the literature. This procedure also allowed computation of Cronbach's alphas, treating each target word-ill minus target word-healthy as a test item, which requires complete data. Across the data set, 7.9% of all possible responses were imputed; at least 1 invalid response (of 16 possible) occurred in 83% of participants.)

## 3. Results

Based on procedures used in similar previous research [20, 22, 29] participants were excluded from analyses when the error rate exceeded 40%. Based on these criteria, three participants were excluded. Internal consistency demonstrated sufficient reliability (*α* = .60), leaving 29 participants for analyses. These values are consistent with other implicit attitude measures reported in the literature.

The mean Tired GNAT *D* score (M = 2.05; SD = 4.06) was significantly different from zero, (*t*(28) = 2.72; *p* = .011) indicating that, on average, tired words were associated with illness. The means (standard deviations) for the measures and subscales included in this study were as follows: TSS = 16.65 (8.93), VSS = 10.66 (4.47), PSS = 6.00 (5.18), and RRS = 5.31 (2.51). In other studies, mean TSS of 10.0 and 30.1 have been reported for unselected university students (*N* = 179) and diagnosed SAD patients coming for light treatment for the first time (*N* = 122) [13].

**Figure 1:**
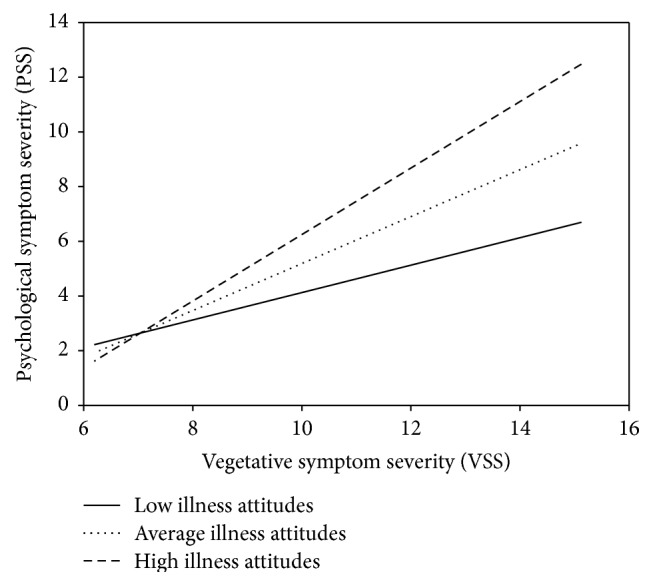
Illness attitudes to fatigue moderate the VSS-PSS relationship.

To test the hypothesis that the relationship between the severity of vegetative symptoms and the severity of psychological symptoms is moderated by implicit attitudes, a moderated regression was conducted, in which VSS scores, GNAT scores, and their product predicted PSS scores (Table 1). The overall regression was significant (*R* ^2^ = .620; *p* < .001) and consistent with the hypothesis; the interaction was statistically significant (*p* = .017), indicating that the strength of the relationship between VSS and PSS varied as a function of the GNAT scores. To interpret the interaction, simple slopes were calculated based on model parameter values. As can be seen in Figure 1, psychological symptom severity was positively associated with vegetative symptom severity at high, medium, and low levels of GNAT score (mean and ±1 SD; all three *p* values ≤ .012). However, the greater the illness-related implicit attitudes toward fatigue were the stronger this relationship was.

The final hypothesis was that a negative, illness-related interpretation of symptoms is positively associated with a ruminative response style. Consistent with the hypothesis, GNAT scores were significantly positively associated with rumination:* r*(28) = .39 and *p* = .01. That is, individuals with stronger illness-related negative implicit attitudes towards fatigue also tended to have a more ruminative response style.

## 4. Discussion

The aims of the present study were (1) to develop an implicit measure of illness attitudes toward the commonly experienced seasonal vegetative symptom of fatigue and (2) to use this implicit measure to examine the role of implicit attitudes in the contexts of the Dual Vulnerability Model of seasonal depression and response style theory. Evidence suggests that the implicit measure was successful in that the average GNAT score was significantly greater than zero and that the GNAT scores were correlated as expected with other variables. Such implicit tests may be less prone to bias and effects of social desirability and limited insight.

Results supported the hypothesis that implicit illness attitudes about fatigue moderated the relationship between vegetative symptom severity and psychological symptom severity. This finding is consistent with work by Young and colleagues [10] and Whitcomb-Smith and colleagues [8] demonstrating that vegetative symptoms interact with cognitive vulnerabilities to predict seasonal cognitive/affective symptoms. The hypothesis that implicit illness attitudes towards vegetative seasonal symptoms are associated with rumination was also supported.

The findings suggest that implicit measures such as the GNAT can provide an important source of information which may facilitate a deeper understanding of attitudes associated with seasonal symptomatology and perhaps other psychopathologies. Additionally, this study provides further support for a cognitive vulnerability to SAD as described by the Dual Vulnerability Model of seasonal depression [9, 10]. This study also provides evidence that a ruminative response style is associated with more negative illness attitudes toward the seasonal vegetative symptom of fatigue. The passive, repetitive nature of ruminative thoughts may make negative cognitions (such as illness attitudes) more easily accessible. Future experimental research, such as stimulating ruminative thinking in a laboratory setting, may help explain how ruminative thinking affects implicit cognition.

The implicit attitude measures in this study assessed the degree to which the participants interpreted the vegetative symptom of fatigue as “illness-related.” However, it is very possible that other types of interpretations could be involved in a cognitive vulnerability towards developing a seasonal episode. For example, some individuals may have a focus on the egodystonic nature of the symptoms or the degree to which they consider the symptoms to impair their functioning [30]. By adjusting attribute categories, future GNATs could be used to assess what other types of attitudes are associated with seasonal symptoms. In the case of this study, it should be kept in mind that these attitudes may be symptom-specific.

Continuing to develop a greater understanding of the cognitions and thinking styles associated with mood symptoms in the wintertime can help inform existing therapeutic interventions [30]. For example, if dysfunctional attitudes increase for both those with SAD and healthy individuals in the fall (as described above) [16], perhaps cognitive distortions and rumination maintain these dysfunctional attitudes in individuals with SAD. Implicit attitude research may also shed some light on which attitudes tend to lead to worsening of symptoms and therefore are most beneficial to target in therapy. Although this study focused in particular on attitudes toward physical symptoms associated with seasonal depression, the development of the implicit tests used in this study may be useful in elucidating the relationship between attitudes towards other physical changes and psychological symptoms. For example, similar measures examining implicit attitudes towards symptoms of specific diseases or pain may be useful for health psychologists. In this way, implicit measures may help to shed light on problematic attitudes and cognitions for a variety of disorders.

These results need to be understood in the context of the limitations of the study. The size of the sample was small and participants were university students. Therefore, it is important that the study be replicated with larger samples and with clinical samples. In addition, the data were cross-sectional. A stronger test of the hypotheses would come from longitudinal data which could make stronger causal inferences about the impact of implicit attitudes on the interpretation of vegetative symptoms.

## 5. Conclusion

According to the Dual Vulnerability Model [10], seasonal affective disorder is the result of the tendency to negatively appraise physiological changes that occur in the wintertime, which leads to an increase in cognitive and mood symptoms. This study developed a method to assess the appraisal of vegetative changes as indicating illness using the GNAT [22], an implicit associations test. We hypothesized that implicit attitudes would interact with the vegetative symptom of fatigue to predict the severity of cognitive symptoms and that rumination would be associated with stronger negative attitudes towards seasonal symptoms. Results showed that illness attitudes toward fatigue moderated the relationship between vegetative and cognitive symptoms. Rumination also predicted illness-related implicit attitudes towards fatigue. The study provides support for the role of negative appraisals of vegetative symptoms in the development of cognitive symptoms in seasonal depression. Results are also consistent with the idea that rumination might contribute to the strength of these negative appraisals.

## Figures and Tables

**Table 1:** Moderated regression analysis for illness attitudes (GNAT *D*) moderating vegetative symptom severity (VSS) predicting psychological symptom severity (PSS).

Model	*B*	SE	*t*	*p*	CI_95_
Constant	5.750	.636	9.047	<.001	4.441, 7.059
VSS	8.58	.147	5.844	<.001	.556, 1.161
GNAT *D*	.319	.170	1.875	.073	−.031, .669
VSS *∗* GNAT *D*	.088	.034	2.552	.017	.017, .159

*N* = 29. *R* ^2^ = .620, *F* (3,25) = 13,582, and *p* < .001.

## Appendix

### Word Stimuli Used in the Tired GNAT

*Tired Stimuli.* The following words were used: Fatigue, Drowsy, Sluggish, Exhausted, Low-Energy, Drained, Weary, and Zonked.

*Healthy Stimuli.* The following words were used: Well, Healthful, Strong, Vital, Fit, Lively, Able-Bodied, and Thriving.

*Ill Stimuli.* The following words were used: Sick, Ailing, Unwell, Diseased, Infected, Hospitalized, Feverish, and Germy.

*Neutral Distractor Stimuli.* The following words were used: Noisy, Copper, Chair, Radio, Pencil, Square, Slippery, Shoe, Month, Dog, Rug, Bookshelf, Tiny, Jewelry, Rapid, Fluffy, Violet, and Dusty.

*Hungry Distractor Stimuli.* The following words were used: Famished, Cravings, Munchies, Want Sweets, Sweet-Tooth, Starving, Eat More, and Ravenous.
